# Supplementary material for: Automatic generation of complementary auxiliary basis sets for explicitly correlated methods
Source: J Comput Chem. 2022 Jul 19;43(25):1690–700. doi: 10.1002/jcc.26970 (PMC9544771; doi:10.1002/jcc.26970)
Supplement: Supplementary file 1 — Table S1. Average percentage of discarded functions for the W4‐08 species in MP2‐F12 Table S2. Composition of autoCABSs generated from VnZ‐F12 orbital basis sets Table S3. Composition of autoCABSs generated from AVnZ‐F12 orbital basis sets Table S4. Average contribution (%) to the total wall clock times in CCSD‐F12b of four large species Table S5. Calculated frequencies (cm–1) of C2H2 using autoCABSs in CCSD(T)(F12*)/VDZF12 vs. YP'S VDZ‐F12/OptRI and SH's VDZ‐F12/OptRI+ as CABS Table S6. Calculated frequencies (cm–1) of C2H2 using various autoCABSs in CCSD(T)‐F12b/VDZ‐F12 vs. YP'S VDZ‐F12/OptRI and SH's VDZ‐F12/OptRI+ as CABS Table S7. Calculated frequencies (cm–1) of C2H2 using various autoCABSs in MP2‐F12/VDZ‐F12 vs. YP'S VDZ‐F12/OptRI and SH's VDZ‐F12/OptRI+ as CABS [file JCC-43-1690-s001.pdf]

Supporting information for

Automatic generation of complementary auxiliary basis sets (CABS) for explicitly correlated methods.

Emmanouil Semidalas[1] and Jan M.L. Martin[1]

[1]Department of Molecular Chemistry and Materials Science, Weizmann Institute of Science, 7610001, Reḥovot, Israel. Email: gershom@weizmann.ac.il

Contents

Instructions for using the autoCABS program.. S1

Table S1. Average percentage of discarded functions for the W4-08 species in MP2-F12. .. S2

Table S2. Composition of autoCABSs generated from VnZ-F12 orbital basis sets............... S3

Table S3. Composition of autoCABSs generated from AVnZ-F12 orbital basis sets............ S4

Table S4. Average contribution (%) to the total wall clock times in CCSD-F12b of four large species. S5

Table S5. Calculated frequencies (cm^{-1}) of C_2H_2 using autoCABSs in CCSD(T)(F12*)/VDZ-F12 vs. YP'S VDZ-F12/OptRI and SH's VDZ-F12/OptRI+ as CABS.................................. S6

Table S6. Calculated frequencies (cm^{-1}) of C_2H_2 using various autoCABSs in CCSD(T)-F12b/VDZ-F12 vs. YP'S VDZ-F12/OptRI and SH's VDZ-F12/OptRI+ as CABS............... S6

Table S7. Calculated frequencies (cm^{-1}) of C_2H_2 using various autoCABSs in MP2-F12/VDZ-F12 vs. YP'S VDZ-F12/OptRI and SH's VDZ-F12/OptRI+ as CABS................. S6

Instructions for using the autoCABS program

This Python implementation generates an auxiliary-complementary basis set (autoCABS) and is available at Github (see https://github.com/msemidalas/autoCABS).

The geometrically averaged exponents, the diffuse and tight functions plus two tight p exponents for the p-block elements, and two additional layers of exponents are added to the autoCABS when the cardinal number of the orbital basis set is D. For larger orbital basis sets, n = T, Q, 5, the program similarly adds the geometrically averaged exponents, the diffuse functions, tight functions without additional p exponents, and one single layer of exponents to the generated autoCABS.

For input instructions, you use a basis set file in an ORCA or MOLPRO format for one element at a time, and name it as either ‘Element.orca’ or ‘Element.mpro’ where element refers to the atomic symbol, (for the atom of carbon, e.g., c.orca or c.mpro).
Make sure that you place the three files, the autoCABS generation script, the my_functions.py with the function definitions, and the orbital basis set file (either in an Orca or Molpro format) in a single folder.

Then to generate an autoCABS for n = D, simply type
python3 AutoCABS-generation-geom_mean_tight_extended_2p_and_diffuse_plus_two_layers.py

To generate an autoCABS from a larger orbital basis set with a cardinal number n larger than D,
python3 AutoCABS-generation-geom_mean_tight_and_diffuse_plus_single_layer.py

The autoCABS is exported in four different formats, MOLPRO, ORCA, TURBOMOLE, and Psi4.

Following the procedure described above, the autoCABS can be generated for the other elements and then all of them should be combined into a single file, there is a simple bash script that assists in that.

Table S1. Average percentage of discarded functions for the W4-08 species in MP2-F12.

CABS		%		
	n =	D	T	Q
VnZ-F12/autoCABS0		0	0	0.050
VnZ-F12/autoCABS0$^+$		0	0	0.036
VnZ-F12/autoCABS0$^-$		0	0	0.175
VnZ-F12/autoCABS0$^\pm$		0	0	0.141
VnZ-F12/autoCABS1$^+$		0	0	0.033
VnZ-F12/autoCABS1$^\pm$		0	0.0005	0.141
VnZ-F12/autoCABS2$^\pm$		0	0.0004	0.139
AVnZ-F12/autoCABS0$^\pm$		0	0.0298	0.441
AVnZ-F12/autoCABS1$^\pm$		0	0.0139	0.257
AVnZ-F12/ autoCABS2$^\pm$		0	0.0272	0.274

Table S2. Composition of autoCABSs generated from VnZ-F12 orbital basis sets.

	Primitive orbital basis	autoCABS0	autoCABS0^{+}	autoCABS0^{-}	autoCABS0$^{\pm}$	autoCABS1$^{\pm}$	autoCABS2$^{\pm}$
	VDZ-F12			VDZ-F12-autoCABS variants			
H	5s2p	2s1p	3s2p	3s2p	4s3p	4s3p2d	4s3p2d1f
He	7s2p	3s1p	4s2p	4s2p	5s3p	5s3p2d	5s3p2d1f
Li-Be	12s6p2d	3s4p1d	4s5p2d	4s5p2d	5s6p3d	5s6p3d2f	5s6p3d2f1g
B-Ne	11s6p2d	3s4p1d	4s5p2d	4s5p2d	5s6p3d	5s6p3d2f	5s6p3d2f1g
Na	17s13p3d	3s4p2d	4s5p3d	4s5p3d	5s6p4d	5s6p4d3f	5s6p4d3f2g
Mg-Ar	16s13p3d	3s4p2d	4s5p3d	4s5p3d	5s6p4d	5s6p4d3f	5s6p4d3f2g
	VTZ-F12			VTZ-F12-autoCABS variants			
H	6s3p1d	3s2p2d	4s3p3d	4s3p3d	5s4p4d	5s4p4d3f	5s4p4d3f2g
He	8s3p1d	4s2p2d	5s3p3d	5s3p3d	6s4p4d	6s4p4d3f	6s4p4d3f2g
Li-Ne	13s7p3d2f	4s5p2d1f	5s6p3d2f	5s6p3d2f	6s7p4d3f	6s7p4d3f2g	6s7p4d3f2g1h
Na	20s14p4d2f	4s5p3d1f	5s6p4d2f	5s6p4d2f	6s7p5d3f	6s7p5d3f2g	6s7p5d3f2g1h
Mg-Ar	17s14p4d2f	4s5p3d1f	5s6p4d2f	5s6p4d2f	6s7p5d3f	6s7p5d3f2g	6s7p5d3f2g1h
	VQZ-F12			VQZ-F12-autoCABS variants			
H	8s4p2d1f	4s3p1d1f	5s4p2d2f	5s4p2d2f	6s5p3d3f	6s5p3d3f2g	6s5p3d3f2g1h
He	9s4p2d1f	5s3p1d1f	6s4p2d2f	6s4p2d2f	7s5p3d3f	7s5p3d3f2g	7s5p3d3f2g1h
Li-Ne	15s9p4d3f2g	5s6p3d2f1g	6s7p4d3f2g	6s7p4d3f2g	7s8p5d4f3g	7s8p5d4f3g2h	7s8p5d4f3g2h
Na-Mg	21s15p5d3f2g	5s6p4d2f1g	6s7p5d3f2g	6s7p5d3f2g	7s8p6d4f3g	7s8p6d4f3g2h	7s8p6d4f3g2h
Al-Ar	21s13p5d3f2g	5s6p4d2f1g	6s7p5d3f2g	6s7p5d3f2g	7s8p6d4f3g	7s8p6d4f3g2h	7s8p6d4f3g2h
	V5Z-F12			V5Z-F12-autoCABS variants			
H	10s5p3d2f1g	5s4p2d1f1g	6s5p3d2f2g	6s5p3d2f2g	7s6p4d3f3g	7s6p4d3f3g2h	7s6p4d3f3g2h
He	11s5p3d2f1g	6s4p2d1f1g	7s5p3d2f2g	7s5p3d2f2g	8s6p4d3f3g	8s6p4d3f3g2h	8s6p4d3f3g2h
B-Ne	17s11p5d4f3g2h	6s7p4d3f2g1h	7s8p5d4f3g2h	7s8p5d4f3g2h	8s9p6d5f4g3h	8s9p6d5f4g3h	8s9p6d5f4g3h
Al-Ar	22s15p6d4f3g2h	6s6p5d3f2g1h	7s7p6d4f3g2h	7s7p6d4f3g2h	8s8p7d5f4g3h	8s8p7d5f4g3h	8s8p7d5f4g3h

Table S3. Composition of autoCABSs generated from AVnZ-F12 orbital basis sets.

	Primitive orbital basis	autoCABS0$^{+}_{-}$	autoCABS1$^{+}_{-}$	autoCABS2$^{+}_{-}$
	AVDZ-F12	AVDZ-F12-autoCABS variants		
H	5s3p	4s4p	4s4p3d	4s4p3d2f
B-Ne	11s6p3d	5s6p4d	5s6p4d3f	5s6p4d3f2g
Al-Ar	16s12p4d	5s6p5d	5s6p5d4f	5s6p5d4f3g
	AVTZ-F12	AVTZ-F12-autoCABS variants		
H	6s4p2d	5s5p3d	5s5p3d2f	5s5p3d2f1g
B-Ne	13s7p4d3f	6s7p5d4f	6s7p5d4f3g	6s7p5d4f3g2h
Al-Ar	17s13p5d3f	6s7p6d4f	6s7p6d4f3g	6s7p6d4f3g2h
	AVQZ-F12	AVQZ-F12-autoCABS variants		
H	8s5p3d2f	6s6p4d3f	6s6p4d3f2g	6s6p4d3f2g1h
B-Ne	15s9p5d4f3g	7s8p6d5f4g	7s8p6d5f4g3h	7s8p6d5f4g3h
Al-Ar	21s13p6d4f3g	7s8p7d5f4g	7s8p7d5f4g3h	7s8p7d5f4g3h

Table S4. Average contribution (%) to the total wall clock times in CCSD-F12b of four large species.

OBS (VnZ-F12)	CABS	SCF	3-index transformation in F12 part	CCSD iterations	DF-MP2-F12	F12b
D	VnZ-F12/ autoCABS2$^{\pm}$	9.5	14.1	68.4	5.6	2.4
T		12.0	18.1	64.9	2.4	2.6
Q		15.2	33.0	48.7	0.9	2.2
D	VnZ-F12-OptRI	9.2	14.1	69.0	5.3	2.4
T		11.9	18.1	65.2	2.0	2.7
Q		15.2	32.9	48.9	0.7	2.3
D	VnZ-F12-OptRI+	9.6	13.9	68.1	6.1	2.4
T		12.1	18.1	65.0	2.2	2.6
Q		15.1	33.6	48.3	0.7	2.2
D	VnZ-F12-MP2Fit	9.2	13.8	68.6	6.0	2.4
T		11.9	18.1	64.8	2.6	2.6
Q		15.1	33.2	48.4	1.1	2.2
D	VnZ-F12-JKFit	9.1	13.6	67.3	7.6	2.3
T		12.0	18.2	64.5	2.6	2.7
Q		15.1	33.3	48.4	1.0	2.2
D	reference-OptRI	2.3	2.4	12.0	82.9	0.4
T		7.0	10.8	38.2	42.5	1.5
Q		13.6	30.9	44.1	9.4	2.0

Table S5. Calculated frequencies (cm^{-1}) of C_2H_2 using autoCABSs in CCSD(T)(F12*)/VDZ-F12 vs. YP'S VDZ-F12/OptRI and SH's VDZ-F12/OptRI+ as CABS.

Auto CABS0	Auto CABS0$^+$	Auto CABS0$^-$	Auto CABS0$^\pm$	Auto CABS1$^+$	Auto CABS1$^\pm$	auto CABS2$^\pm$	auto CABS2$^\pm$ + extend. 1p	auto CABS2$^\pm$ + extend. 2p	YP's VDZ-F12-OptRI	SH's VDZ-F12-OptRI+
520.54	494.14	494.14	421.8	616.11	620.68	596.14	600.41	600.24	619.01	618.64
520.54	494.14	494.14	421.8	616.11	620.69	596.14	600.41	600.25	619.01	618.64
741.83	740.48	740.48	736.14	753.82	752.65	754.63	754.64	754.71	751.44	751.05
741.83	740.48	740.48	736.14	753.82	752.65	754.63	754.64	754.71	751.44	751.06
2015.51	2015.27	2015.27	2015.55	2013.43	2013.77	2013.09	2013.44	2013.55	2013.16	2013.63
3406.67	3406.41	3406.41	3406.95	3413.89	3413.61	3412.34	3412.6	3412.65	3413.79	3413.57
3501.24	3500.85	3500.85	3501.37	3507.04	3506.53	3505.42	3505.72	3505.78	3507.38	3506.80

Table S6. Calculated frequencies (cm^{-1}) of C_2H_2 using various autoCABSs in CCSD(T)-F12b/VDZ-F12 vs. YP'S VDZ-F12/OptRI and SH's VDZ-F12/OptRI+ as CABS.

Auto CABS0	Auto CABS0$^+$	Auto CABS0$^-$	Auto CABS0$^\pm$	Auto CABS1$^+$	Auto CABS1$^\pm$	auto CABS2$^\pm$	auto CABS2$^\pm$ + extend. 1p	auto CABS2$^\pm$ + extend. 2p	YP's VDZ-F12-OptRI	SH's VDZ-F12-OptRI+
586.91	583.57	583.57	533.26	617.09	614.51	640.82	604.16	604.00	609.96	609.48
586.91	583.57	583.57	533.26	617.10	614.52	640.85	604.17	604.01	609.97	609.61
749.31	749.08	749.08	745.49	751.17	748.98	771.54	750.74	750.81	747.26	746.72
749.31	749.08	749.08	745.49	751.17	748.99	771.54	750.74	750.81	747.2	746.73
2009.11	2008.85	2008.85	2009.09	2006.77	2007.86	2051.80	2007.43	2007.55	2007.12	2007.67
3405.41	3405.18	3405.18	3405.96	3410.14	3410.29	3429.88	3409.99	3410.03	3410.87	3410.97
3499.04	3498.68	3498.68	3499.33	3502.52	3502.52	3525.98	3501.98	3502.03	3503.74	3503.53

Table S7. Calculated frequencies (cm^{-1}) of C_2H_2 using various autoCABSs in MP2-F12/VDZ-F12 vs. YP'S VDZ-F12/OptRI and SH's VDZ-F12/OptRI+ as CABS.

Auto CABS0	Auto CABS0$^+$	Auto CABS0$^-$	Auto CABS0$^\pm$	Auto CABS1$^+$	Auto CABS1$^\pm$	auto CABS2$^\pm$	auto CABS2$^\pm$ + extend. 1p	auto CABS2$^\pm$ + extend. 2p	YP's VDZ-F12-OptRI	SH's VDZ-F12-OptRI+
610.56	605.20	605.20	557.35	636.80	632.95	617.60	621.89	621.70	630.99	630.45
610.56	605.20	605.20	557.36	636.81	632.95	617.60	621.89	621.79	630.99	630.54
758.49	758.21	758.21	754.82	759.66	757.25	760.01	760.06	760.12	756.97	756.31
758.49	758.21	758.21	754.82	759.66	757.25	760.01	760.06	760.13	756.97	756.32
1981.73	1981.48	1981.48	1981.68	1979.12	1980.27	1979.70	1980.08	1980.20	1979.77	1980.32
3439.37	3439.13	3439.13	3439.68	3443.44	3443.63	3442.98	3443.24	3443.29	3444.20	3444.32
3528.31	3527.94	3527.94	3528.39	3531.13	3531.15	3530.59	3530.90	3530.96	3532.64	3532.38
